# The relation of Bleomycin Delivery Efficiency to Microbubble Sonodestruction and Cavitation Spectral Characteristics

**DOI:** 10.1038/s41598-020-64213-y

**Published:** 2020-05-08

**Authors:** Martynas Maciulevičius, Mindaugas Tamošiūnas, Mindaugas S. Venslauskas, Saulius Šatkauskas

**Affiliations:** Biophysical Research Group, Faculty of Natural Sciences, Vytautas Magnus University, Vileikos g. 8, Kaunas, Lithuania

**Keywords:** Biophysics, Cell death

## Abstract

The concurrent assessment of principal sonoporation factors has been accomplished in a single systemic study. Microbubble sonodestruction dynamics and cavitation spectral characteristics, ultrasound scattering and attenuation, were examined in relation to the intracellular delivery of anticancer drug, bleomycin. Experiments were conducted on Chinese hamster ovary cells coadministered with Sonovue microbubbles. Detailed analysis of the scattering and attenuation temporal functions culminated in quantification of metrics, inertial cavitation dose and attenuation rate, suitable for cavitation control. The exponents, representing microbubble sonodestruction kinetics were exploited to derive dosimetric, microbubble sonodestruction rate. High intracorrelation between empirically-attained metrics defines the relations which indicate deep physical interdependencies within inherent phenomena. Subsequently each quantified metric was validated to be well-applicable to prognosticate the efficacy of bleomycin delivery and cell viability, as indicated by strong overall correlation (R^2^ > 0.85). Presented results draw valuable insights in sonoporation dosimetry and contribute towards the development of universal sonoporation dosimetry model. Both bleomycin delivery and cell viability reach their respective plateau levels by the time, required to attain total microbubble sonodestruction, which accord with scattering and attenuation decrease to background levels. This suggests a well-defined criterion, feasible through signal-registration, universally employable to set optimal duration of exposure for efficient sonoporation outcome.

## Introduction

Sonoporation is a novel therapeutic modality, which exploits microbubbles (MBs) in combination with direct ultrasound (US) application to facilitate the passage of membrane-impermeable bioactive agents, mainly, anticancer drugs and foreign DNA into cells and tissues^1–5^. US-MB mediated drug delivery research is primarily focused to advance sonoporation as efficient cancer treatment strategy. Research works have reported successful sonoporation applications to amplify the level of cytolethality, imposed by various anticancer drugs, like bleomycin^1,4,6–8^ (BLM), doxorubicin^9–11^ and cisplatin^12,13^ with the aim to kill malignant cells *in vitro* and conjointly reduce tumour volume *in vivo*.

Cell sonoporation process is initiated by cavitating MBs, directly exposed to US irradiation. MB cavitation is generally divided into two distinctive patterns: stable and inertial^6,7,14–16^. Stable cavitation is associated with periodic MB expansion and contraction around equilibrium volume. The frequency spectrum of US waves emitted by stably cavitating MBs contains harmonic, subharmonic and ultraharmonic components. Inertial cavitation occurs at higher acoustic pressures when MBs rapidly grow in size and violently collapse. This results in US field that has components in broad frequency range, causing a phenomenon called “broadband noise” to emerge^17–20^. Cavitating MBs create physical phenomena such as microstreaming^14,21–23^ and liquid jets^24–26^ that induce mechanical shear forces^21,27,28^ resulting in transient cell membrane permeabilisation by initiating formation of pores as well as endocytotic mechanisms^2,3,29–31^.

The amount of acoustic energy, subjected to cells ought to be accurately measured and strictly regulated if the desirable level of cell permeabilisation is to be achieved while decreasing violent secondary aftermaths triggered by inertial cavitation, essentially, significant cell death. Thus, the development of uniform and precise cavitation dosimetry model, allowing to accurately control and monitor the extent of cavitation, is of the utmost importance.

There are a lot of parameters influencing sonoporation efficiency, such as MB physical properties, cell type, characteristics of the medium or the molecules, selected for intracellular delivery^3,32,33^. However, the physical characteristics (center frequency, acoustic pressure, duty cycle, duration, etc.) of US excitation are on the primary focus. The parameters of US excitation that are subjected to the experimental system have been referred to as the input parameters by Hallow *et al*.^34^. Since US input parameters only indirectly influence sonoporation efficiency, the sonoporation dosimetric research is mainly concentrated on the study of the ultrasonic signals emitted by cavitating MBs.

Passive cavitation detection (PCD) is the most sensitive and advanced technique for sonoporation dosimetry. It exploits passive US transducer, positioned to monitor the direct US signal emitted by cavitating nuclei^35–38^. Early investigations involving PCD were mostly focused on bubble inertial cavitation threshold detection and subsequent method validation^35–37^. The fundamental research by Everbach *et al*.^39,40^ was the first to present quantitative proof about MB scattering signal feasibility for the prognostication of biological effect. The authors have calculated average root mean square (RMS) values of US scattered by MBs and discovered that RMS strongly correlated with hemolysis as well as platelet sonolysis. Chen *et al*. have advanced the field of implicit dosimetry by proposing the metric, inertial cavitation dose (ICD), defined as the integral of RMS of scattered US signal cumulated in exposure duration scale^38,41^. The latter was used to prognosticate erythrocyte hemolysis in a variety of physical conditions applied. Subsequent studies, performed by different researchers, have shown ICD to be a suitable dosimetric for intracellular delivery of calcein^34^, doxorubicin^10^ and DNA^16,20,42^, calcein extraction^43^ and cell viability^16,20,34,38,43^ as well as the prognostication of pore size^16^. Subesequent approaches were performed to monitor cavitation activity in gel tunnels^44,45^, through mouse bone scull^46^, in perfused rabbit ear vessels^47^ as well as create images of cavitation intensity spatial distribution^48,49^. More advanced biophysical research were designed to quantify ICD *in vivo* and relate it to the damage of rabbit blood vessel endothelial cells^50,51^ as well as to determine the cavitation threshold for blood brain barrier disruption in mice^52^, rabbits^53^ as well as non-human primates^54,55^. In addition to this, Zhou *et al*. have demonstrated that intracellular transmembrane current was directly associated to the onset of MB broadband noise emissions at a single cell level, using *Xenopus* oocytes^56^.

US wave decreases in amplitude when it propagates through the suspension containing MBs. This phenomenon is termed US attenuation and can be successfully exploited to monitor MB cavitation behavior^57,58^. US attenuation measurements have been successfully applied to evaluate MB sonodestruction dynamics^1,59,60^, determine the acoustic characteristics of different MB types^61–63^, relate sonoporation efficiency to the attenuation level^64^ as well as to determine the resonant frequency of MBs^58,65^.

Tamosiunas *et al*. have presented MB sonodestruction rate, a metric based on MB concentration measurements, and defined as the rate constant of MB concentration decay exponent^7^. This metric was suitable to prognosticate bleomycin^7^ and doxorubicin^10^ delivery efficiency *in vitro* as well as cell death^7^.

Despite the current advances in sonoporation research, the relations between spectral estimates, MB concentration and molecular delivery in both PNP and exposure duration scales still remain poorly understood. Even though there are a lot of different studies in the field of sonoporation dosimetry, to our knowledge, there is no research, where: MB concentration, US scattering, US attenuation and molecular delivery efficiency measurements were performed at the same experimental conditions. Thus, we present a combined and detailed research where MB scattering and attenuation signals as well as MB sonodestruction are monitored in relation to BLM delivery efficiency and cell viability. All the factors that characterise sonoporation were evaluated in a single study in both pressure and exposure duration domains of the acoustic field by using double-transducer PCD system. In addition to this, we propose a criterion to determine optimal exposure duration for sonoporation, based on MB scattered US signal registration. We believe that this comprehensive research will improve the current understanding within the interdependencies between MB cavitation estimates and molecular delivery as well as draw detailed quantitative connections between them.

## Results

### Cavitation experimental results

#### MB sonodestruction

**Figure 1:**
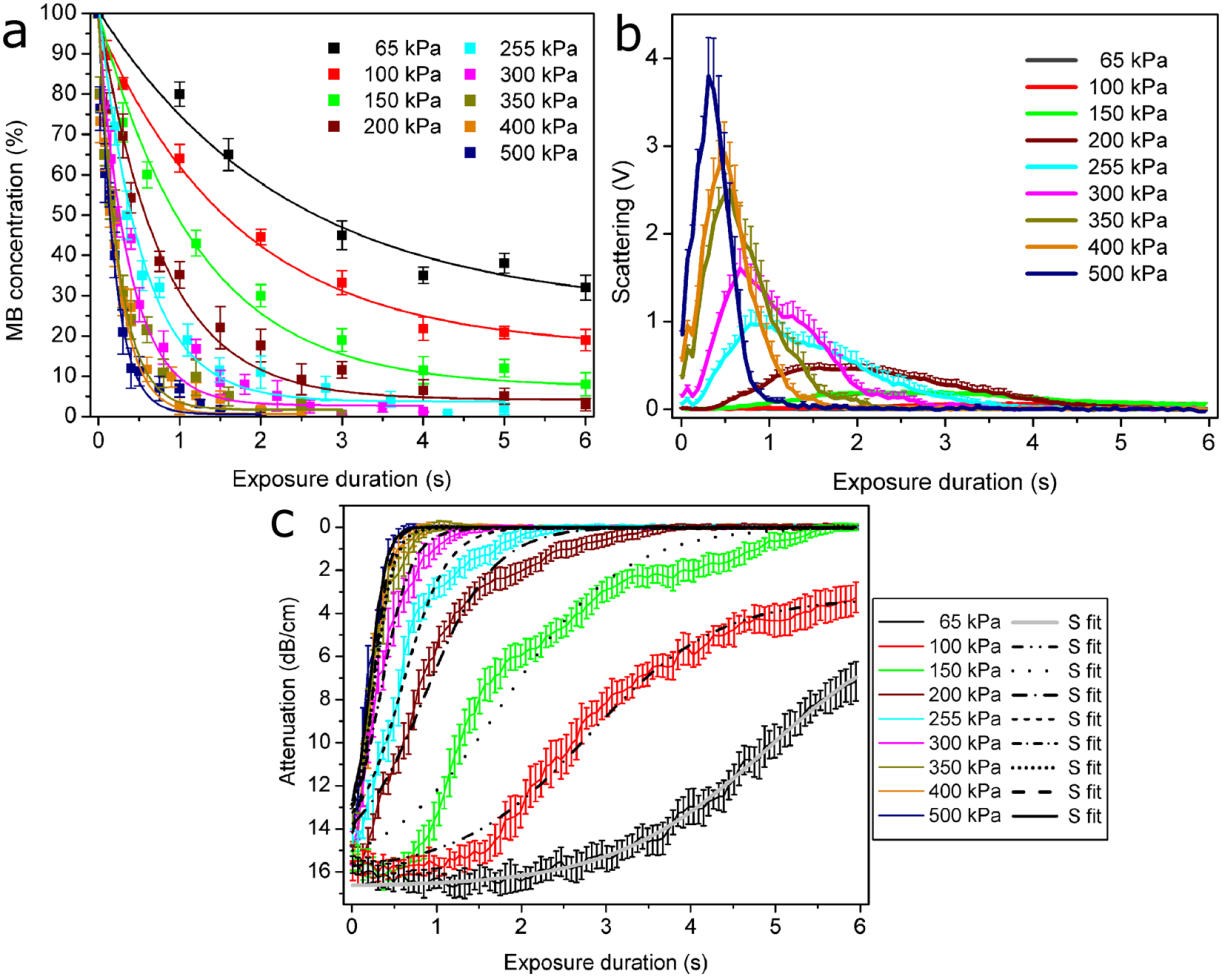
MB concentration measurement results in exposure duration scale at different acoustic pressures (**a**). Scattering results in exposure duration scale at different acoustic pressures (**b**). Attenuation results in exposure duration scale at different acoustic pressures **(c**). Experimental conditions: 1 MHz center frequency, 1 kHz pulse repetition frequency, 10% duty cycle (100 μs on, 900 μs off) US, 6 s exposure duration; 1.36 × 10^7^ MBs/ml; data represent the mean ± SEM of *n* = 4 experimental replicates.

Spectrophotometric MB assay showed exponential MB concentration decay over time at different PNP values (Fig. 1a). At lower acoustic pressures, MB decay occurs slower with exponents having lower rates. As PNP increases, MB concentration decreases faster in exposure duration scale with exponents having higher rates. This shows that at higher acoustic pressures MB inertial cavitation activity starts and proportionally finishes earlier. The complete MB sonodestruction (~0%) is achieved earlier with increasing PNP values.

#### US scattering

US signals, scattered by MBs, were quantified as RMS values in 1.5–1.75 MHz frequency range. Scattering curves at different PNP values are shown in Fig. 1b. These curves have highly expressed rising, peak and falling parts, corresponding to increase, maximum and decrease of MB cavitation activity. PNP value increase results in stronger MB cavitation activity and corresponding higher scattering amplitudes that is observed earlier in the scale of exposure duration. This implies that higher acoustic pressures evoke maximal MB cavitation in shorter time. In respect, scattering decrease to background value (0 V) is achieved earlier with PNP increase.

#### US attenuation

MB induced US attenuation of the exciting US signal was evaluated as the logarithmic ratio of spectral RMS in 0.9–1.1 MHz frequency domain. The results showed attenuation curves to be similar to sigmoidal curves (Fig. 1c). At increasing PNP, the attenuation curves become steeper and decrease to 0 dB/cm faster. This is in agreement with MB sonodestruction and US scattering results and likewise indicate that higher PNP values evoke early-starting MB cavitation (MB concentration decrease), which correspondingly lasts shorter.

### Sonoporation experimental results

#### Sonoporation in exposure duration scale

**Figure 2:**
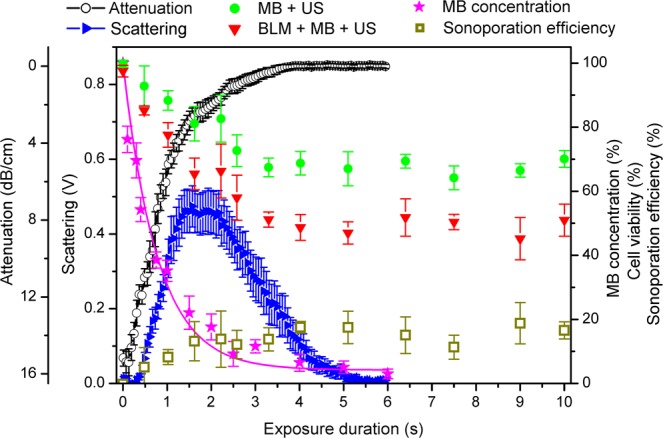
MB sonodestruction, scattering, attenuation, cell viability and sonoporation efficiency in dependence on 200 kPa PNP US exposure duration. The percentage of sonoporation efficiency was evaluated as the difference in cell death: (BLM + MB + US) − (MB + US) − (BLM + MB), (BLM + MB) is (BLM + MB + US) group at 0 s exposure duration. Experimental conditions: 1 MHz center frequency, 1 kHz pulse repetition frequency, 10% duty cycle (100 μs on, 900 μs off) US, 6 s exposure duration; 1.36 × 10^7^ MBs/ml, 0.8 × 10^6^ cells/ml, 20 nM BLM concentration; data represent the mean ± SEM of *n* = 4 experimental replicates.

**Figure 3:**
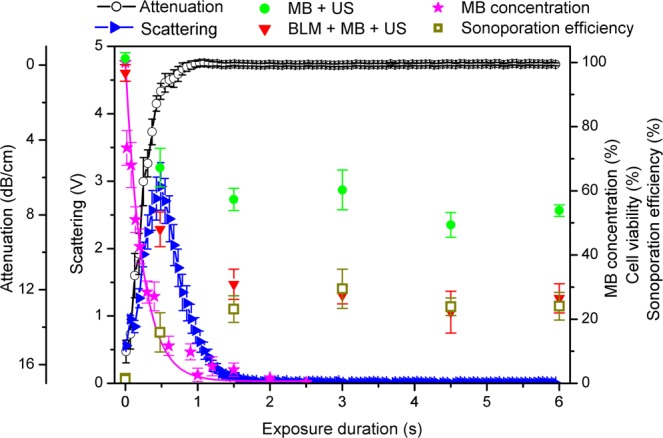
MB sonodestruction, scattering, attenuation, cell viability and sonoporation efficiency in dependence on 400 kPa PNP US exposure duration. The percentage of sonoporation efficiency was evaluated as the difference in cell death: (BLM + MB + US) − (MB + US) − (BLM + MB), (BLM + MB) is (BLM + MB + US) group at 0 s exposure duration. Experimental conditions: 1 MHz center frequency, 1 kHz pulse repetition frequency, 10% duty cycle (100 μs on, 900 μs off) US, 6 s exposure duration; 1.36 × 10^7^ MBs/ml, 0.8 × 10^6^ cells/ml, 20 nM BLM concentration; data represent the mean ± SEM of *n* = 4 experimental replicates.

BLM delivery efficiency and cell viability temporal dynamics were evaluated at 200 and 400 kPa PNP. At 200 kPa PNP the scattering curve is wide, MB concentration decay rate is slow and, correspondingly, attenuation decrease curve is slow (Fig. 2). Conversely, at 400 kPa PNP value the scattering curve is narrow and MB concentration as well as attenuation decrease are fast (Fig. 3). Figures 2 and 3 also plot the combined results of three different groups: cavitation (MB + US), therapeutic (BLM + MB + US) and sonoporation efficiency. The sonoporation efficiency was evaluted as the difference in cell death between different groups: (BLM + MB + US) − (MB + US) − (BLM + MB)^6,7^.

The dynamics of four main sonoporation processes: MB concentation decrease, scattering, attenuation and sonoporation efficiency are presented in Figs. 2 and 3. MB concentration decay occurs simultaneously with attenuation decrease and cell viability decrease as well as sonoporation efficiency increase. All these processes occur within the scattering curve and reach their corresponding saturation levels about the time when scattering decreases to background level. It can be clearly observed that the saturation margin of all three sonoporation groups approximately coincides with the margin of complete MB sonodestruction as well as attenuation and scattering decrease to background levels.

Two different acoustic pressures were selected in order to show that sonoporation groups reach their corresponding plateau values earlier at 400 kPa than at 200 kPa PNP. Therefore, MB concentration, scattering and attenuation faster decrease to complete MB destruction and background levels at 400 kPa than at 200 kPa acoustic pressure. In addition to this, it was suitable to show the gradual dynamics of cell viability and sonoporation efficiency within wide scattering curve, at 200 kPa acoustic pressure.

The results, presented in Figs. 2 and 3, imply the following: it is unnecessary to continue US excitation after the estimates decrease to the background levels as there are no changes in either sonoporation group, and the most importantly “Sonoporation efficiency” group. Additional US irradiation could only induce harmful effects to cells. This indicates the optimal exposure duration for sonoporation to coincide with the decrease of any estimate, MB concentration, scattering or attenuation, to background level.

We have previously shown that additional cell irradiation with US after complete MB sonodestruction led only to additional cell death increase without increasing sonoporation efficiency^6^. The cell-killing effect of US alone was enhanced as US parameters corresponding to higher cavitation activity were applied, at 0.88 MHz center frequency, 100% duty cycle, 500 kPa PNP US additional cell death occured within 3 s of exposure duration after complete MB sonodestruction had been achieved.

#### Sonoporation in PNP scale

For the temporal optimization of sonoporation efficiency, we have chosen the criterion of scattering decrease to background level as an indicator for maximal sonoporation efficiency, while sustaining high cell viability. This is because MB scattering signals can be successfully monitored in various experimental conditions: *in vitro*^10,16,34,38–43,56^, *ex vivo*^46–48^ and *in vivo*^50–55^.

For this reason, we have determined the approximate exposure duration that scattering decreases to background margin (~0 V) at different PNPs, except for 65–150 kPa where the end of scattering curve was not observed due to limited recording capabilities of our hardware. For these PNP values we have chosen 6 s duration. Thus, the exposure duration values applied for optimised sonoporation experiments are: 65 kPa–6 s, 100 kPa–6 s, 150 kPa–6 s, 200 kPa – 5.1 s, 255 kPa – 4.02 s, 300 kPa – 3 s, 350 kPa – 2.04s, 400 kPa – 1.5 s and 500 kPa – 1.2 s.

**Figure 4:**
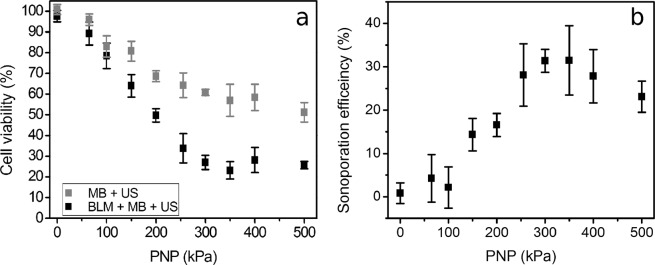
The results of sonoporation experiments in PNP scale. Cell viability decrease in cavitation (MB + US) and therapeutic (MB + BLM + US) groups after sonoporation (**a**). The percentage of sonoporation efficiency (**b**), evaluated as the difference in cell death: (BLM + MB + US) − (MB + US) − (BLM + MB), (BLM + MB) is (BLM + MB + US) group at 0 kPa PNP. Experimental conditions: 1 MHz center frequency, 1 kHz pulse repetition frequency, 10% duty cycle (100 μs on, 900 μs off) US, 6 s exposure duration; 1.36 × 10^7^ MBs/ml, 0.8 × 10^6^ cells/ml, 20 nM BLM concentration; data represent the mean ± SEM of *n* = 4 experimental replicates.

The cell viability and sonoporation efficiency dependence on PNP after optimisation are given in Fig. 4a,b, respectively. With increasing acoustic pressure cell viability decreases in both (MB + US) and (BLM + MB + US) groups. (BLM + MB + US) group decreases fast up to ~34% (at 255 kPa) and then reaches plateau, while (MB + US) group decreases fast up to ~57% (at 350 kPa) and at higher acoustic pressures decreases only slightly.

The sonoporation efficiency was evaluted as it is described in the methodical section. The obtained optimised percentage of BLM delivery efficiency is shown in Fig. 4b. It can be seen that BLM delivery increase occurs up to ~350 kPa with the achieved sonoporation efficiency of ~31% and ~57% cell viability. At further increase in acoustic pressure, sonoporation efficiency slightly decreased.

The lowest cell viability in cavitation group, ~51%, was observed at 500 kPa PNP. The control group of (BLM + MB) showed ~98% cell viability, indicating no impact of BLM, MBs or their combination to the cells.

### Metrics

**Figure 5:**
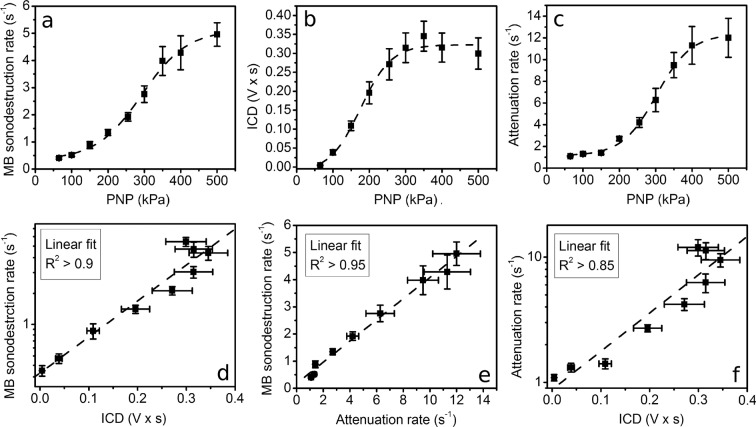
Sonoporation metrics: MB sonodestruction rate (**a**), ICD (**b**) and attenuation rate (**c**). Correlation between metrics: MB sonodestruction rate and ICD (**d**), MB sonodestruction rate and attenuation rate (**e**), attenuation rate and ICD (**f**). Experimental conditions: 1 MHz center frequency, 1 kHz pulse repetition frequency, 10% duty cycle (100 μs on, 900 μs off) US, 6 s exposure duration; 1.36 × 10^7^ MBs/ml; data represent the mean ± SEM of *n* = 4 experimental replicates.

MB concentration results (Fig. 1a) as well as spectral estimates, scattering (Fig. 1b) and attenuation (Fig. 1c), were used to calculate sonoporation metrics, MB sonodestruction rate (Fig. 5a), ICD (Fig. 5b) and attenuation rate (Fig. 5c).

Both MB sonodestruction rate and attenuation rate directly reflect the speed of MB concentration and US attenuation decrease, respectively. Both curves have sigmoidal dose-response shape.

ICD was calculated for each PNP value during the applied exposure duration. The shape of ICD curve in PNP range similarly to previous estimates is sigmoidal.

All three metrics were tested for their interdependencies (Fig. 5d–f). The correlation results indicate that all three processes are strongly interconnected (R^2^ > 0.85). Faster MB concentration decrease is directly reflected by faster attenuation decrease and similarly induces higher scattering amplitudes, defining higher ICD values.

### Correlation analysis

**Figure 6:**
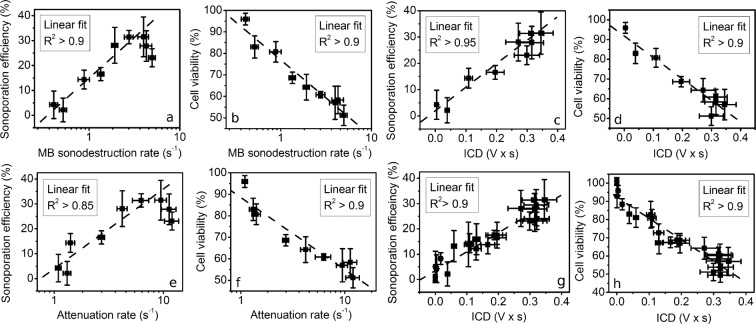
Correlation between metrics and sonoporation efficiency as well as cell viability in cavitation (MB + US) group. MB sonodestruction rate and BLM delivery effiency (**a**) and cell viability (**b**); attenution rate and BLM delivery effiency (**c)** and cell viability (**d**); ICD and BLM delivery effiency (**e**) and cell viability (**f**); pooled ICD and BLM delivery effiency (**g**) and cell viability (**h**). Experimental conditions: 1 MHz center frequency, 1 kHz pulse repetition frequency, 10% duty cycle (100 μs on, 900 μs off) US, 6 s exposure duration; 1.36 × 10^7^ MBs/ml, 0.8 × 10^6^ cells/ml, 20 nM BLM concentration; data represent the mean ± SEM of *n* = 4 experimental replicates.

Each metric was tested for the ability to prognosticate sonoporation efficiency and cell viability in cavitation (MB + US) group (Fig. 6). All three metrics, MB sonodestrction rate, ICD and attenuation rate, had strong correlation (R^2^ > 0.85) with BLM delivery efficiency (Fig. 6a,c,e) and cell viability (Fig. 6b,d,f), obtained in PNP range. The approximation for BLM delivery efficiency was performed up to 350 kPa, where the percentage of BLM delivery was increasing. Cell viability was approximated in whole PNP range.

The metric, ICD, conversely to time-dependent metrics, is integral measure, thus, it was possible to evaluate ICD prognostication abilities for pooled data, that is, for BLM delivery efficiency and cell viability results from both PNP and exposure duration ranges. The approximation for pooled data was performed in whole scale for both BLM delivery efficiency (Fig. 6g) and cell viability (Fig. 6h). The correlation between pooled ICD and BLM delivery efficiency as well as cell viability, similarly to previous, was strong (R^2^ > 0.9).

## Discussion

In the current research we have used PCD system, composed of two passive US receivers, positioned at 90° and 180° angles to the transmitter, in order to register side-scattered and attenuated US signals, respectively. Earlier studies, performed by Chen *et al*., Lai *et al*., Hallow *et al*. and Qiu *et al*., exploited single-transducer PCD system, complemented only for scattering registration^16,20,34,38,41^. Previous sonoporation studies^1,6,7,64,65^ have applied active cavitation detection techniques in order to monitor US, attenuated by MBs. Conversely to their approaches, our passive attenuation detection alowed us to avoid possible secondary impact on MBs.

To the extent of our knowledge, there is no similar systematic study to evaluate MB concentration decrease, attenuation, scattering and molecular delivery to cells. Following our previous study^10^ where we monitored MB concentration, US scattering and doxorubicin delivery, we performed a detailed study evaluating three aforementioned sonoporation factors in relation to BLM delivery efficiency in both exposure duration and acoustic pressure domains. Other researchers have evaluated molecular delivery only with either scattering^16,20,34^ or attenuation^64^ or MB concentration decay^6,7^.

MB concentration dynamics, US scattering, US attenuation and bioeffect efficiency data show the relevance of the duration of exposure, required to obtain total MB sonodestruction. By that time BLM delivery and cell viability values have attained their respective saturation levels. Subsequent US irradiation neither had impact on BLM delivery efficiency nor on cell viability. In addtion to this, the time for complete MB sonodestruction approximately coincides with both attenuation and scattering decrease to background values. This notion implies that MB concentration, attenuation or scattering can be interchangeably used to monitor MB dynamics during US application. Moreover, this implies that attenuation or scattering can be exploited to optimise sonoporation in the exposure duration scale. Previously, we have shown that the adjustment of US exposure parameters resulting in higher US energy delivered to cells results in additional cell viability decrease, induced by exposure to US alone, after complete MB destruction had already been achieved^6^. Thus, complete MB destruction or scattering/attenuation decrease to background level may be exploited to prognosticate optimal duration of exposure in order to attain high sonoporation efficacy herewith sustaining sufficient level of cell viability. Because the evaluation of attenution for *in vivo* or clinical practice is hard due to shadowing effect^66^, it is much easier and more feasible to register side-scattered or backscattered US waves. The proposed optimisation criterion– the monitoring of scattering decrease to background level – can be well applied for *in vivo* studies as MB scattering signals can be easily monitored during *in vivo* experiments^50–55^. In addition to this, we have quantified scattering in 1.5–1.75 MHz frequency band. As lower frequency US is less attenuated in the tissue environment, this frequency range can be exlpoited in order to precisely calculate RMS values for *in vivo* dosimetric applications.

The delay between the attenuation and scattering decrease to background levels, is due to small MB amount left that is still able to scatter US, but is too weak to inhibit the attenuation signal. In addition to this, similar scattering values are obtained at times corresponding to high and low MB concentrations (for 200 kPa PNP, these are at ~1 s and ~3 s, respectively (Fig. 2)). This is due to shadowing effect^66^: outer MBs obscure the signal coming from MBs located in the deeper layers of the cuvette. Thus, part of scattering is lost from the detection. As MB concentration during US exposure decreases, scattering of previously shadowed MBs gets detected. As a consequence, scattering values obtained with higher MB concentration are similar to those obtained while having lower MB concentration.

Karshafian *et al*. and Rahim *et al*. have explored wide spectrum of US input parameters with the aim to determine optimal conditions for sonoporation^67,68^. However, their empirically determined optimal parameters were inherent to their specific experimental conditions, mainly, due to the exploited experimental setup and the range of US parameters tested. Conversely to their studies, we propose output parameters for sonoporation efficiency optimisation that are prime characteristics of MB behaviour, directly affecting cells and specific sonoporation results.

Uncontrolled MB cavitation can do severe damage, implying the importance of real-time cavitation control. The analysis of MB concentration, US scattering and US attenuation data has culminated in quantitatively evaluated metrics: MB sonodestruction rate, ICD and attenuation rate, which have analogous dose-response shaped tendency and strong intercorrelation (R^2^ > 0.85), implying well-defined interrelations among representative fundamental phenomena. Main purpose of metric comparison is to show that dosimetrics, derived after US signal analysis, are in agreement with the kinetics of MB concentration in a respective manner. When acoustic pressure is raised, MB destruction curves recede more quickly, scattering temporal functions augment to greater magnitudes, ICD increases, in accordance, attenuation curves decrease faster. ICD is the integral metric that includes the time when MBs are active as well as the amplitude, associated to the intensity of their cavitation activity, while attenuation rate and MB sonodestruction rate are directly time-dependent metrics. Thus, we have determined the relation between metrics that are different in nature.

ICD is the most conventional measure, used for dosimetric purposes in sonoporation, and it is experimentally-approved according to reliable prediction of calcein^34^, doxorubicin^10^, DNA^16,20^ transfer as well as calcein release^43^ efficiency. MB sonodestruction rate was proved to be suitable for the prognostication of BLM^7^ and doxorubicin^10^ delivery efficiency. Escoffre *et al*. have shown maximal efficacy of exogenous DNA uptake to be obtained in coadminitration with Vevo Micromarker MBs, that were characterised by rapid US attenuation decrease^64^. Previously we have shown, that calcein release as well as cell viability decrease were associated to the speed of attenuation decrease^43^. In this study we have quantified the metric, attenuation rate, which similarly to other metrics had strong correlation (R^2^ > 0.85) with BLM delivery efficiency as well as cell viability. The latter metrics can be interchangeably used to prognosticate BLM sonotrasnfer or cell viability decrease during sonoporation.

However, the most popular metric, ICD, is a relative measure, as it incorporates the absolute values of scattered signal RMS, which are dependent on particular sonoporation equipment, conditions and computational algorithms, used by different research groups. This brings about the difficulties to relate concrete absolute ICD values to specific *in vitro*/*in vivo* bioeffects^10,38,51^. The variety of sonoporation dosimetry studies have been performed at different biophysical conditions and experimental setups beginning from artifical phantoms *in vitro*^38–45^, leading to complex approaches *ex vivo*^46–48^ and *in vivo*^50–55^ and even fortified by cavitation intensity spatial mapping^48,49,69,70^. As the level of the complexity of experimental sytems advances from simplified to modern, in accordance, the diversity of equipment increases and is accompanied by the development of more intricate analytic methods that become more distinct among the research groups in the field. Therefore, as the setups are improved, the fundamental aspect of universal result reproducibility is missed. The key achievement in this field would be the development of universal dosimetry model allowing to reproduce the results, reported by different research groups. Thus, the time-dependent metrics are more appropriate as they are neither determined by the absolute values of the monitored quantity nor dependent on sonoporation equipment used by the researchers, implying the reproducibility of the results on the universal scale^7,10^. Therefore, our study is a systemic research, which after concurrent assessment of principal sonoporation factors in relation to biological efficiency, provides the insights valuable for the development of universal sonoporation dosimetry system. It is achieved by addressing and providing empirical insights in time-dependent metrics and their application for BLM delivery optimisation and prognostication. The proposed sonoporation optimisation criterion, scattering decrease to baseline, is also time-dependent, therefore, can be employed universally. In general, our findings can be adjusted and subsequently transposed to more state-of-the-art setups, upgraded with cavitation spatial mapping appliances. However, when integral signal from the whole volume, containing MBs, is sufficent to provide necessary information about MB behavior and predict experimental outcome, cavitation signal can be monitored using elementary technique, also reducing the cost of the research. In this case our insights and metrics may be applied by the researchers in more straightforward fashion.

The results obtained in our study imply inertial cavitation to be the key mechanism in cell membrane permeabilization, mainly due to inefficient drug delivery obtained at lower acoustic pressures (up to 100 kPa). However, even at these acoustic pressures, MB concentration was decreasing, however, to a lower extent when compared to higher pressures. In addition to this, our findings demonstrate that MB sonodestruction rate (the direct estimate of MB concentration decrease) strongly correlates with ICD and attenuation rate. Therefore, it can be assumed that inertial cavitation is the main mechanism for molecular delivery in our study. However, the role of stably cavitating MBs, which generate shear stress and, consequently, induce cell membrane permeabilisation^14,15,68^, increases as oscillating MBs appear in close proximity to cells, as in the case they are positioned or targetted^14,15,21^. Thus, cell membrane deformation, induced by microstreaming, presumably, can activate endocytotic processes^3,71^ as well as pore formation^3,14,71^. Increasing distances between MBs and cells diminish the effects of microstreaming, especially in suspension conditions, where MBs and cells are stochasticly moving. In those conditions, inertial cavitation seems to be the key mechanism for efficient sonoporation^6,7,16,20,34^ as schockwaves, microjets and other phenomena, associated to inertial cavitation^24–26,28^, take over.

In overall, in this study we have presented a complete summarized single research, which presents three estimates that characterise MB behavior, evaluated in relation to anticancer drug delivery efficiency to cells. The current study summarizes previous works by Chen *et al*., Lai *et al*., Qiu *et al*., Escoffre *et al*., Tamosiunas *et al*., Maciulevicius *et al*. and others^6,7,16,20,34,38–43^. We believe that findings, obtained in this research, improve the current understanding in sonoporation mechanism, advance the field of sonoporation dosimetry and reveal new opportunities for method development.

## Materials and methods

### Cell line

Chinese hamster ovary (CHO) cell culture was cultivated in DMEM (Sigma-Aldrich, MO, USA) supplemented with 10% fetal bovine serum (Sigma-Aldrich, MO, USA), 1% L-glutamine (Invitrogen Inc., Carlsbad, CA, USA) and 100 U/mL penicillin with 10 μg/mL streptomicyn (Sigma-Aldrich, MO, USA). The cells were grown as monolayers in 96 mm culture dishes (TPP, Trasadingen, Switzerland), incubated at 37 °C, in a humidified atmosphere with 5% CO_2_. The cells were harvested with trypsin/EDTA solution (Sigma-Aldrich, MO, USA), after trypsin inactivation with the growth medium; the cells were resuspended in 1 × PBS (Lonza Inc., Rockland, ME, USA)^43^.

### Experimental setup

**Figure 7:**
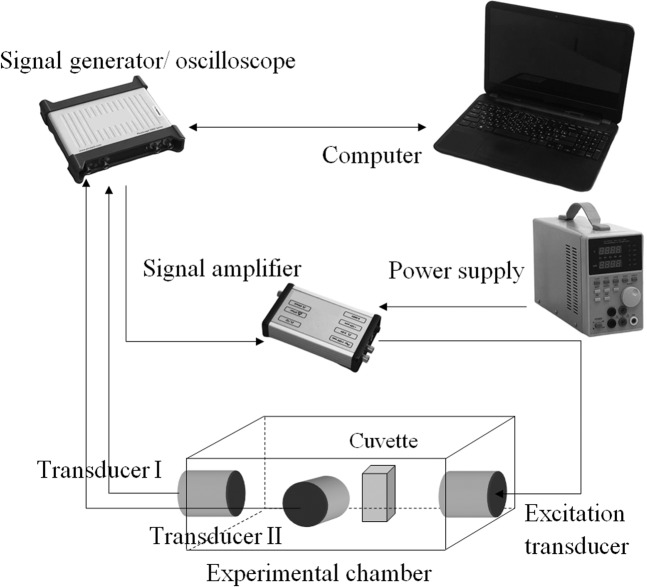
Experimental setup used for cavitation and sonoporation experiments.

The experimental setup used for both MB cavitation and sonoporation experiments is shown in Fig. 7. The system is composed of experimental chamber, US signal generation and acquisition hardware^10,43^. The arbitrary waveform generator/ oscilloscope (Picoscope 5242B, Picotech, Cambridgeshire, UK) was used both to deliver and record US signals. The electric signals were amplified by lab-made high frequency signal amplifier (Kaunas University of Technology, Kaunas, Lithuania), powered by high voltage power supply (MCP Lab Electronics, Shanghai, China). The unfocused transducer (Medelkom, Kaunas, Lithuania), 18 mm diameter, 1 MHz center frequency 0.9 − 1.2 MHz, −6 dB bandwidth, was used for MB excitation.

Passive cavitation detection (PCD) system was designed using transducers I and II. Transducer I – 10 mm diameter, 1 MHz center frequency 0.1–2.2 MHz, −27 dB bandwidth transducer (Doppler Electronic Technologies, Guang Zhou, China) positioned in line with the excitation transducer. Transducer II – 6 mm diameter, 10 MHz center frequency 1–15 MHz, −27 dB bandwidth transducer (Doppler Electronic Technologies, Guang Zhou, China) set at the right angle to the excitation transducer.

The sonoporation cuvette (Plastibrand, Wertheim, Germany) during both cavitation and sonoporation experiments was filled with 1 mL of MB suspension or MB and cell suspension, depending on the experiment type. The distances between the center of the cuvette and the excitation transducer, transducer I, transducer II are 1 cm, 5.8 cm, 3.2 cm, respectively.

The experiments were performed at room temperature (24 °C). US acoustic pressure calibration was performed using needle hydrophone (HNR-1000; Onda Corp, Sunnyvale, CA, USA) with an active element of 1 mm diameter. The hydrophone’s tip was placed in the cuvette after removing cuvette’s distal wall. Thus, the determined US acoustic pressure corresponds to real *in situ* conditions. In order to diminish the impact of standing waves to the experimental samples the inner surface of the experimental chamber was ligned with acoustic absorber (AptFlex F28, Precision acoustics, Dorchester, UK).

### Cavitation experiments

#### Microbubble sonodestruction evaluation

SonoVue MBs (Bracco Diagnostics Inc., Geneva, Switzerland) were prepared according to the manufacturer’s instructions.

MB concentration was evaluated using optical method, described previously^10^. The method is based on the correlation between MB concentration evaluated using hematocytometer (Assistent, Sondheim, Germany) and MB suspension optical density values. During experiments the cuvette was filled to 1 ml volume. Final MB concentration was estimated to be 1.36 × 10^7^ MBs/ml. New portion of MB suspension was used for each experimental point. For each experimental repetition MB concentration was defined as a ratio of optical density after to before US exposure. This was performed with the aim to diminish the influence of MB self-destruction to the experimental results.

MB sonodestruction mesurements were performed after sample exposure to 1 MHz center frequency, 10% duty cycle (100 μs on, 900 μs off), and 65, 100, 150, 200, 255, 300, 350, 400, 500 kPa peak negative acoustic pressures (PNP) US.

#### Cavitation signal quantification

With the aim to quantify MB cavitation activity, MB cavitation signals were recorded using transducers I and II. Signals acquired using transducer I correspond to attenuated US signals, while using transducer II – to scattered US signals.

The experiments of MB cavitation signal recording were performed without cells with the aim to avoid any possible MB and cell interaction^20^. The signals were evaluated for both groups with MBs (+MB group) and for the background group without MBs (−MB group). The cuvette was filled with 1 ml PBS for background signal recording. Due to the limitations of our signal recording hardware scattering and attenuation signals were recorded separately.

The cavitation signals of overall 6 s exposure duration were recorded in 100 frames (10 ms each), at 31.25 MS/s sampling rate, 8 bits resolution. Therefore, overall 6 s exposure duration resulted in 1 s of recorded exposure duration. Since the discretisation in frames is sufficiently fast (discretisation period is 60 ms) compared to MB decay dynamics, the influence of the data loss to the estimates, due to signal recording, is negligible.

**Figure 8:**
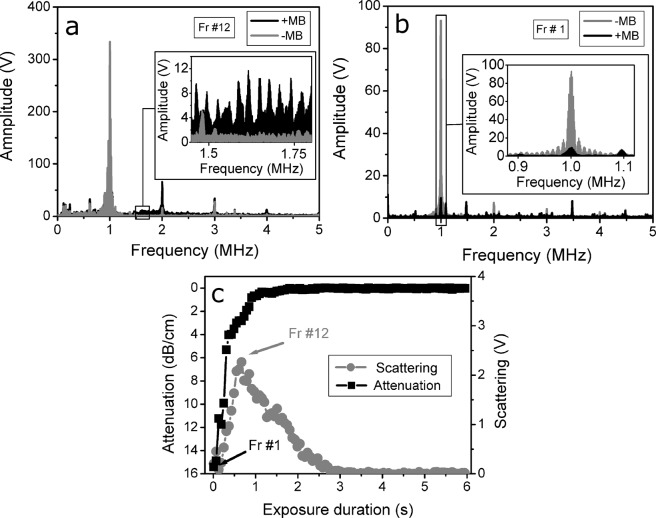
The FFT values of pulses from frame #12 of the scattered US signal (**a**) and frame #1 of atenuated US signal (**b**) contributing to the highest scattering and attenuation values (**c**). Note the inverse scale of attenuation (dB/cm). Experimental conditions: 1 MHz center frequency, 1 kHz pulse repetition frequency, 10% duty cycle (100 μs on, 900 μs off) US, 6 s exposure duration; 1.36 × 10^7^ MBs/ml concentration.

The recorded signals were transformed to frequency spectra using fast Fourier transform (FFT) for root mean square (RMS) calculation. In order to ensure the stability of the quantified estimates, the US signals from the whole frame were transformed to frequency domain. RMS was calculated in frequency spectrum:

1 $$RMS=\sqrt{\frac{1}{n}({x}_{1}^{2}+{x}_{2}^{2}+\ldots +{x}_{n}^{2})},$$

where *n* is the number of values in the frequency spectrum, obtained after FFT; *x* – is the amplitude value, associated to particular frequency value (*n*). Figure 8a represents the FFT spectrum of scattered US signal of +MB and −MB groups, recorded at 300 kPa US excitation. The highest difference between +MB and –MB groups was determined in 1.5–1.75 MHz range, represented in the 12th frame. Thus, this range was chosen for RMS calculation for scattered US waves, as described previously^10,43^. Background RMS was subtracted from the RMS, obtained from experimental groups with MBs present. This resulted in differential RMS of scattered signal and reduced the background influence to the “scattering” estimate.

Figure 8b shows the FFT spectrum of the attenuated US pulses, recorded in the 1st frame at 300 kPa. The range for RMS calculation of attenuated signals was selected around transmitting frequency −0.9–1.1 MHz, because this range was associated to the highest attenuation as it is indicated in the Fig. 8b, similarly as described previously^43^. The logarithmic ratio of RMS without MBs to RMS with MBs was evaluated as a measure of MB induced US attenuation.

Figure 8c represents both scattering and attenuation estimates, plotted in the exposure duration scale. It is observed that scattering develops above 0 V and then decreases to 0 V (background level), while the attenuation starts way above 0 dB/cm and decreases to 0 dB/cm (background level).

### Metric quantification

#### MB sonodestruction rate

In order to obtain MB sonodestruction rate MB concentration decay curves were approximated with exponential function^7^:

2 $$MB\,concentration(t)=A{e}^{-\alpha t}+B,$$

where *A* is the amplitude of the exponential function; *B* is the offset; *α* is the rate constant of the exponential function; *t* is time. *α* defines the rate of MB concentration decay and is termed MB sonodestruction rate. 1/ *α* results to time constant of the exponent function, *τ*, which implies the exposure duration necessary for MB concentration to decrease to 1/e times (37%) of the initial value.

#### Inertial cavitation dose

In order to obtain inertial cavitation dose (ICD), the integral of scattering vs. exposure duration was calculated^20,38^:

3 $$ICD={\int }_{0}^{{t}_{Final}}\,Scattering(t)dt,$$

where *ICD* is inertial cavitation dose, *t* is time, 0 indicates 0 s (the beginning of the exposure duration), *t*_*Final*_ indicates the exposure duration at which the integration is finished.

The scattering values were cumulated during recorded exposure duration (1 s) at particular PNP as it was described previously^10,43^.

#### Attenuation rate

With the aim to obtain attenuation rate, attenuation curves in exposure duration scale were approximated using sigmoidal function. Similarly, as described by Fan *et al*.^72^:

4 $$Attenuation(t)=\frac{{A}_{1}-{A}_{2}}{1+{e}^{k(t-{t}_{C})}}+{A}_{2},$$

where *A*_1_ and *A*_2_ are the initial and final attenuation values, respectively; *k* is the rate constant of the sigmoidal function; *t* is time; *t*_*C*_ is time at the sigmoidal center value.

The rate of the function, *k*, indicates the steepness of the attenuation curve and is defined as the attenuation rate.

### Sonoporation experiments

Sonoporation experiments were performed using anticancer drug bleomycin (BLM) (Teva, Haarlem, Netherlands).

The experimental groups for BLM delivery efficiency evaluation were divided as follows: 1) control group (cells alone, no US irradiation); 2) cavitation group (cells, MB, with US irradiation), abbr. (MB + US); 3) therapeutic group (cells, BLM, MB, with US irradiation), abbr. (BLM + MB + US).

In the therapeutic group the final volume in the experimental cuvette was 1 ml with final 0.8 × 10^6^ cells/ml, 1.36 × 10^7^ MBs/ml and 20 nM BLM concentrations. In 1 and 2 experimental groups (without MB/BLM) the final 1 ml volume was achieved with PBS administration.

Cell and MB concentrations were evaluated by counting MBs in the hematocytometer (Assistent, Sondheim, Germany) using optical microscope (Nikon Eclipse TS100, Tokyo, Japan). The MB to cell ratio in this research was estimated to be 17:1.

The cells were exposed to 1 MHz central frequency pulsed US at 10% duty cycle (100 µs ON, 900 µs OFF), 65–500 kPa acoustic peak negative pressures (PNP) and for 0–10 s exposure durations at 200 kPa as well as for 0–6 s exposure durations at 400 kPa. The exposure durations for PNP series were selected to coincide with the end of peak of the scattering curve at corresponding particular PNP, except for 65–150 kPa, where the end of scattering curve was not observed within 6 s of signal recording. Thus, PNP and their corresponding exposure duration values were selected as follows: 65 kPa – 6 s, 100 kPa – 6 s, 150 kPa – 6 s, 200 kPa – 5.1 s, 255 kPa – 4.02 s, 300 kPa – 3 s, 350 kPa – 2.04 s, 400 kPa – 1.5 s and 500 kPa – 1.2 s.

Cell viability was evaluated using cell clonogenic assay^43^. After US irradiation, the cells were incubated for 10 min at 37 °C. Then they were diluted in growth medium and 100 μL of the suspension (∼330 of cells) was loaded into 4.1 cm^2^ tissue culture dishes (TPP, Trasadingen, Switzerland) containing 2 mL of growth medium. The cells were allowed to grow for 7 days, then fixed in 1 mL of 96% ethanol for 10 min and stained using crystal violet solution (Sigma-Aldrich, St. Louis, MO, USA), containing 2.3% crystal violet, 0.1% ammonium oxalate and 20% ethanol. The number of cell colonies was assessed using light microscope (MBS-9, LOMO, St. Petersburg, Russia) and then normalized to the control.

Cell death in the (BLM + MB + US) group could be the result of: i) the cell death caused by US induced MB cavitation (MB + US) group, ii) the cells killed by BLM and/or MB (BLM + MB) group and iii) BLM sonotransfer, i.e., facilitated BLM intracellular delivery due to reversible sonoporation resulting in cell death due to intracellular BLM toxicity. To reveal the number of sonoporation efficiency, the percentage of cells killed in the (MB + US) group and (BLM + MB) group (therapeutic group with no US irradiation, corresponding to 0 kPa PNP or 0 s exposure duration) were subtracted from the percentage of cells killed in the (BLM + MB + US) group as it was described previously^6,7^.

### Data analysis

The data are presented as the mean ± standard error of the mean (SEM) of 4 experimental replicates (n = 4). Correlation analysis was used to determine the dependence between the metrics and the sonoporation results, the strength of correlation is defined according to the correlation determination coefficient (R^2^). Data analysis was performed using Matlab (Mathworks, Natick, MA, USA) and Origin (OriginLab Co., Northampton, MA, USA) software.
